# Respiratory regulation and lung volume during aquatic and land‐based exercise in healthy Young adults

**DOI:** 10.14814/phy2.70564

**Published:** 2025-09-29

**Authors:** Daisuke Hoshi, Marina Fukuie, Tsubasa Tomoto, Wenxing Qin, Takashi Tarumi, Jun Sugawara, Koichi Watanabe

**Affiliations:** ^1^ Doctoral Program in Sports Medicine, Graduate School of Comprehensive Human Sciences University of Tsukuba Tsukuba Ibaraki Japan; ^2^ Japan Society for the Promotion of Science Tokyo Japan; ^3^ Human Informatics and Interaction Research Institute National Institute of Advanced Industrial Science and Technology Tsukuba Ibaraki Japan; ^4^ Integrated Research Center for Self‐Care Technology National Institute of Advanced Industrial Science and Technology Tsukuba Japan; ^5^ Institute of Health and Sport Sciences University of Tsukuba Tsukuba Ibaraki Japan

**Keywords:** aquatic exercise, breathing pattern, hemodynamics, lung volume

## Abstract

Elevated hydrostatic pressure during water immersion reduces lung volume and compliance at rest. These alterations may persist during exercise, influencing both the respiratory regulation and lung volume. This study compared respiratory regulation and lung volume between land‐based (LC) and aquatic (AC) cycling matched for oxygen uptake (VO_2_). Ten healthy young adults underwent cycling at low and moderate intensities in both environments. Expiratory gas variables (VO_2_) and respiratory variables (minute ventilation and respiratory rate: V_E_ and RR, respectively) were continuously measured using a breath‐by‐breath gas analyzer system. Ventilatory equivalent for VO_2_ (V_E_/VO_2_) was calculated. Using a spirometry system, expiratory and inspiratory reserve volumes (ERV and IRV, respectively), and tidal volume (V_T_) were measured at rest and at each exercise intensity using inspiratory maneuvers and normalized to forced vital capacity (FVC). Although VO_2_ was matched between conditions (*p* > 0.05), AC resulted in significantly higher V_E_, RR, and consequently V_E_/VO_2_ at moderate intensity. Additionally, ERV was lower and IRV was higher during AC compared with LC across all intensities, while FVCs remained unchanged in both conditions. These findings suggest a potential mechanism by which exercise in an aquatic environment may be more effective than land‐based exercise for training the respiratory system.

## 1 INTRODUCTION

Aquatic exercise is appealing to older adults and individuals with physical limitations, such as arthritis and obesity, due to the buoyancy of water which lessens the gravitational load on the lower limb joints (Alberton et al., 2015; Liebs et al., 2012). Despite being a low‐intensity exercise due to buoyancy, aquatic exercise has been reported to effectively impact the respiratory system. It improves respiratory muscle strength (de Souto Araujo et al., 2012; Gallo‐Silva et al., 2024), thoracic mobility (Gallo‐Silva et al., 2024), and functional capacity (McNamara et al., 2013) in patients with chronic pulmonary disease. However, the mechanisms by which such low‐intensity exercise affects the respiratory system remain poorly understood.

The increased hydrostatic pressure caused by water immersion compresses the thoracoabdominal cavity (Agostoni et al., 1966) and lower extremities, leading to significant physiological effects. This compression enhances venous return, which in turn raises airway resistance (Hoshi et al., 2021) and reduces pulmonary compliance (Castagna et al., 2021). Consequently, lung volumes such as vital capacity and expiratory reserve volume (ERV) decrease during head‐up water immersion (Bondi et al., 1976; Girandola et al., 1977). Importantly, these alterations in respiratory mechanics observed at rest may persist into exercise, influencing both lung volumes and the neural control of breathing.

Some limited studies have shown that aquatic exercise using a stationary bicycle increases respiratory rate (RR) and decreases V_T_ compared to land‐based exercise at the same oxygen uptake (VO_2_) (Ayme et al., 2015; Hoshi et al., 2022; Sheldahl et al., 1987). This alteration in ventilatory response is particularly pronounced during moderate to high‐intensity exercise. However, no studies have evaluated both ventilatory regulation and lung volumes simultaneously. This study investigated the effects of water immersion on respiratory regulation and lung volume during low‐ to moderate‐intensity land‐based (LC) and aquatic (AC) cycling performed at the matched VO_2_. We hypothesized that AC would result in increased ventilation for a similar VO_2_ and alter the pattern of lung volume fluctuation from rest to exercise.

## 2 MATERIALS AND METHODS

### 2.1 Ethical approval

All participants were recruited at the University of Tsukuba and gave written informed consent before participation. This study conformed to the standards set by the latest revision of the *Declaration of Helsinki*, except for registration in a database. All experimental procedures were approved by the Research Ethics Committee of the University of Tsukuba (Tai 022–64).

### 2.2 Participants

This study enrolled 10 healthy young adults (five women). None of them were taking medication or had overt cardiovascular, respiratory, or neuromuscular disease or a history of smoking as assessed by a medical history questionnaire.

#### 2.2.1 Experimental design

Participants visited our laboratory three times at least 72 h apart and performed the following measurements in order: (visit 1) a graded peak VO_2_ (VO_2_peak) test, (visit 2) AC, and (visit 3) LC. VO_2_ measured during AC (visit 2) was used as a reference to match VO_2_ during LC (visit 3). Participants were instructed to fast for a minimum of 3 h and refrain from vigorous exercise, alcohol consumption, and caffeine intake for at least 24 h before each visit. All visits were separated by at least 72 h.

##### VO_2_peak measurement (visit 1)

VO_2_peak was measured to determine the maximal aerobic capacity, which was used to match metabolic rates during AC and LC. VO_2_peak testing was conducted on a semi‐recumbent bike (Corival recumbent, Lobe, BV, Netherlands). VO_2_peak was measured using a modified Astrand protocol (Astrand, 1965). The workload started at 60 W for men and at 40 W for women participants and increased by 20 W every 2 min. VO_2_, VCO_2_, and respiratory exchange ratio (RER) were continuously measured during rest and graded exercise using a breath‐by‐breath expiratory gas analyzer system (Aero monitor, MINATO, Osaka, Japan). During testing, brachial blood pressure, three‐lead electrocardiogram, and heart rate were monitored to assess participants' safety (Bayles, 2023). After the rate of perceived exertion reached 17, the workload was increased by 10 W until two of the following criteria were met: (1) 95% heart rate reserve (95% HRR) calculated from the age‐predicted maximum; 95% HRR = (age‐predicted maximum (i.e., 220—age)—rest HR) × 0.95 + rest HR (Karvonen et al., 1957), (2) RER greater than 1.2, (3) unable to maintain cycling at 60 rpm (within ±5 rpm), or (4) rate of perceived exertion higher than 18. VO_2_peak was defined as the VO_2_ averaged over 30 s during the last stage of testing.

After VO_2_peak testing, participants took a break and practiced the measurements of forced vital capacity (FVC) maneuver and inspiratory capacity (IC) for the actual testing on visit 2 and 3.

##### AC (visit 2)

**FIGURE 1:**
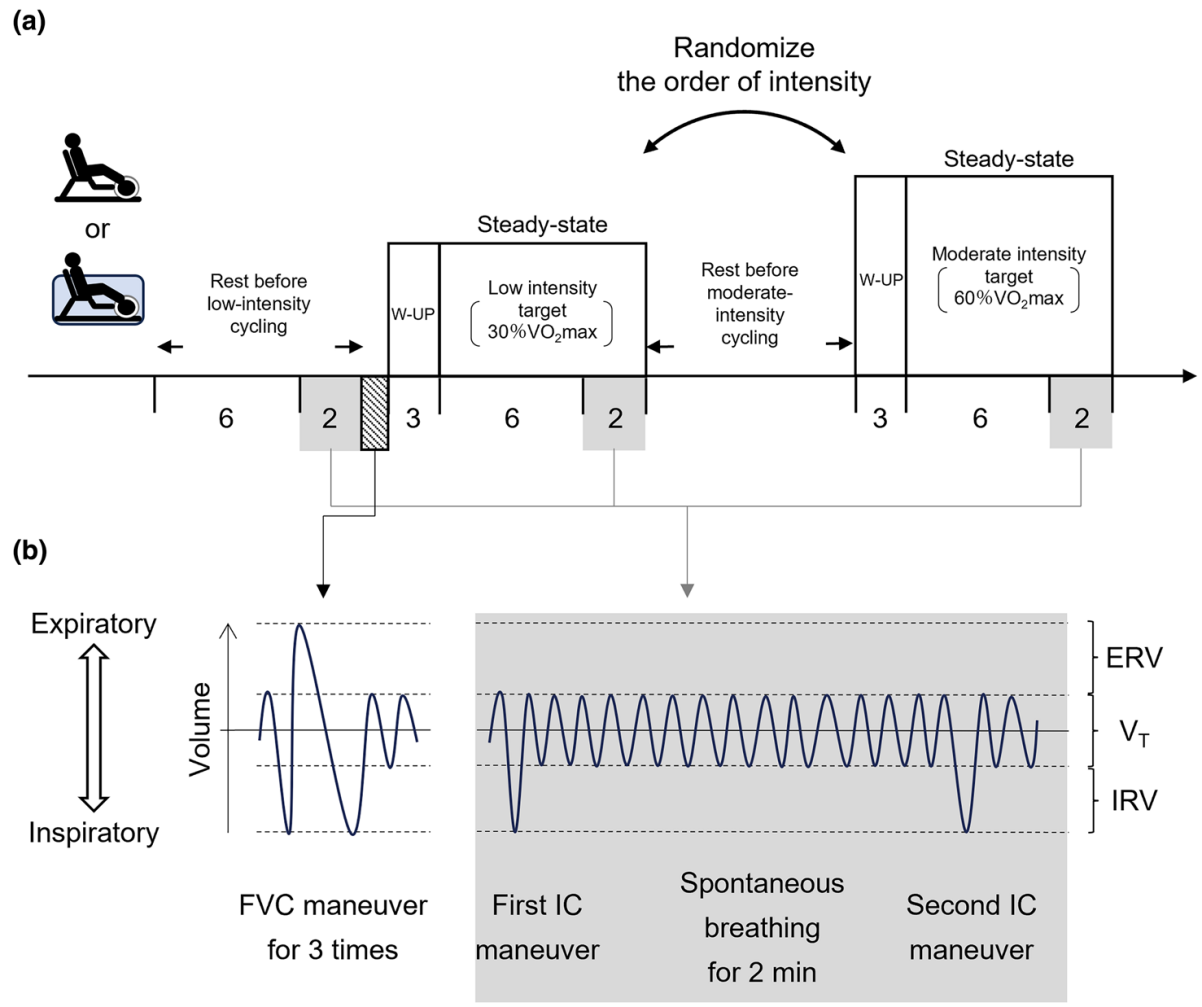
Experimental protocol of aquatic cycling (AC) and land‐based (LC) cycling (a), and timing of FVC and inspiratory capacity (IC) maneuvers (b). Participants rested for 8 min and performed low‐ and moderate‐intensity cycling for 11 min each. During AC, water temperature was thermoneutral (31°C ~ 32°C) and water level was set to the xiphoid appendix of each participant. The gray areas indicate the timing and content of the IC maneuvers. The shaded area indicates the timing of FVC maneuvers (a). IC maneuvers were performed before and after the last 2 min of rest and each cycling and FVC maneuvers were performed after 8‐min first rest (b). ERV, expiratory reserve volume; IC maneuver, inspiratory capacity maneuver; IRV, inspiratory reserve volume; Low, Low‐intensity‐cycling; Moderate, moderate‐intensity cycling; VO_2_, oxygen uptake; V_T_, tidal volume.

Figure 1a shows the experimental protocol of AC and LC. In AC, participants submerged in thermoneutral water (31°C ~ 32°C) which does not affect metabolic rate at rest (Craig Jr. & Dvorak, 1966). The water depth was set to the xiphoid appendix level for each participant. Although this water level did not fully submerge the lungs, previous studies have shown that it is sufficient to increase central blood volume (Carter et al., 2014). Participants performed two intensity cycling exercises in random order: low‐ and moderate‐intensity cycling for 11 min each (including 3‐min warming‐up and 8‐min steady‐state cycling) after 8 min of rest. The cycling intensity was adjusted by the cadence rate. Participants cycled on an aquatic stationary semi‐recumbent bicycle (Hydrorecline, H_3_Oz, Italy) at 40 rpm at low intensity and at 60 rpm at moderate intensity. To prevent the effects of fatigue due to differences in exercise intensity, a break between each exercise intensity was given until all expiratory gas variables returned to the baseline level. The cadence was controlled using a metronome and coaching by the examiner. Throughout AC, three plastic paddles (7 × 22 × 0.5 cm) were attached to the rear wheel to increase the pedaling resistance.

##### LC (visit 3)

Participants performed the LC using an electronic stationary bicycle (Corival recumbent, Lode BV, Netherlands). Similar to visit 2, low‐ and moderate‐intensity cycling was performed in random order (Figure 1a). During 3‐min warm‐up period before each, participants cycled at 40 rpm at low intensity and at 60 rpm at moderate intensity while wattage was adjusted to match VO_2_ with that recorded during each intensity of the AC. In this way, we compared AC and LC with the matched VO_2_ and cadence rate during each intensity of cycling.

To match VO_2_ during LC and AC, the following steps were used. First, the linear regression line between wattage and breath‐by‐breath changes in VO_2_ recorded was calculated during VO_2_peak testing on visit 1. Second, we obtained steady‐state VO_2_ at each intensity during AC on visit 2. Lastly, using both sets of data, the wattage for LC was individually calculated by substituting the VO_2_ measured during AC into the wattage‐VO_2_ regression line estimated on visit 1. VO_2_ was constantly monitored during LC, and wattage was adjusted carefully.

### 2.3 Measurements

#### 2.3.1 Expiratory gas and hemodynamic variables and body temperature

Expiratory gas variables such as VO_2_, %VO_2_peak, and VCO_2_ were continuously recorded by the expiratory gas analyzer system in a breath‐by‐breath manner (Aero monitor, MINATO, Osaka, Japan) during the first 6 min of rest and the first 9 min of cycling in both environments. Data reported are the averages of the first 6 min during rest and 6 min of steady‐state cycling after a 3‐min warming up at each intensity. Tympanic temperature was intermittently measured after the fourth minute during rest and each exercise intensity.

Digital arterial pressure waveform was continuously recorded at the right middle finger by a non‐invasive blood pressure monitor (Human NIBP Nano System, AD Instrument, Colorado Springs, CO, USA) and stored on a computer using a data acquisition system (PowerLab, AD Instrument, Colorado Springs, CO, USA) at the sampling rate of 200 Hz for offline analysis. Participants kept their right hand at heart level throughout resting and cycling. The pressure recording collected using a finger photoplethysmography cuff has been validated against the intra‐arterial recording at rest and during laboratory testing (Parati et al., 1989). There is also good agreement in the evaluation of beat‐to‐beat variations (Lindqvist, 1995). Heart rate and systolic and diastolic blood pressure (SBP and DBP, respectively) were calculated from pulse waveforms, and these blood pressures were corrected by brachial blood pressure. Stroke volume (SV) and cardiac output (CO) were estimated by the Modelflow‐based add‐on program for the data acquisition system. This method has been validated in a variety of conditions including exercise (Sugawara et al., 2003; Wesseling et al., 1993).

#### 2.3.2 Respiratory variables and ventilatory equivalents for VO_2_ and VCO_2_

V_E_, RR, V_T_, and total time per breath (Ttot) were continuously recorded by the expiratory gas analyzer system with a thermal flowmeter in a breath‐by‐breath manner (Aero monitor, MINATO, Osaka, Japan). For data analysis, these variables were averaged over the first 6 min during rest and 6 min of steady‐state cycling after 3 min of warming up at each intensity. To assess respiratory regulation, ventilatory equivalents for VO_2_ and VCO_2_ (V_E_/VO_2_ and V_E_/VCO_2_, respectively) were obtained by dividing V_E_ by VO_2_ and VCO_2_, respectively.

#### 2.3.3 Lung volume variables

Lung volume variables were recorded by a spirometry system (Powerlab, ADInstruments, Colorado Springs, CO, USA) and stored on a computer using a data acquisition system (Powerlab, ADInstruments, Colorado Springs, CO, USA) at the sampling rate of 1 kHz for offline analysis. FVC was measured in water (visit 2) and on dry land (visit 3) after 8 min of rest according to the ATS/ERS guidelines (Miller et al., 2005). Participants performed the IC maneuver twice during another 2 min of rest and each cycling intensity (Figure 1a,b, gray area). To eliminate the drift of volume in the sensing device, the IC maneuvers were performed during the first and last 10 s of the 2‐min cycling exercise, and the volumes were aligned to the ICs (Johnson et al., 1999). V_T_ was averaged for the last 30 s excluding the IC breath, and ERV and IRV were expressed in actual values and calculated as ERV (%FVC) = (FVC—IC)/FVC × 100, IRV (%FVC) = (IC—V_T_)/FVC × 100. The timing of data collection was standardized for all participants. The spirometry system was calibrated before each experiment according to the manufacturer's instructions.

### 2.4 Sample size estimate

Sample size was calculated using the G*Power software. The following values were used according to a previous study (Hoshi et al., 2022) that investigated breathing patterns during exercise in water and on dry land (i.e., effect size: 0.7; α‐level: 0.05; statistical power: 0.95; the number of groups: 2 [LC and AC]; the number of measures: 3 [rest, low, moderate]; considering the refusal of subjects: 2). Therefore, the sample size required for this study was estimated to be 10 participants.

### 2.5 Statistical analysis

Normality of all variables during LC and AC was checked by visual inspection of histograms and the Shapiro–Wilk test. The primary analysis was conducted by linear mixed model based on the intent‐to‐treat principle using all available data (*n* = 10). A three‐way linear mixed model was used to examine the effects of environment (dry land vs. underwater), intensity (low vs. moderate), time (rest vs. exercise), and their interaction (environment × intensity × time). A random intercept and an unstructured covariance model were used to account for within‐individual correlations. In the case of a significant interaction, *post‐hoc* tests were performed to compare between environments at the same intensity with the Bonferroni correction. A paired *t*‐test was used to compare the effects of environments on resting FVC. Estimated marginal means and 95% confidence intervals are reported from the results of linear mixed models, whereas means ± standard deviations are reported from the paired *t*‐test. A *p* < 0.05 was considered statistical significance. All statistical analyses were performed using SPSS Statistics 29.0 for Windows (IBM Inc., Chicago, IL, USA).

## 3 RESULTS

Study participants had a mean age of 25 ± 2 years, height of 167.8 ± 7.3 cm, body mass of 64.2 ± 7.5 kg, body mass index of 22.7 ± 1.3 kg/m^2^, and VO_2_peak of 40.4 ± 5.7 mL/kg/min. Data from one female could not be collected due to a measurement system error.

### 3.1 Expiratory gas and hemodynamic variables and body temperature

Table 1 shows expiratory gas variables and body temperature at rest and during cycling in both environments. Across both environments, low‐intensity cycling was performed at 27%VO_2_peak (95% CI, 23%–31%VO_2_peak) during LC and 27% VO_2_peak (95%CI, 24%–31% VO_2_peak) during AC on average, while moderate‐intensity cycling was performed at 61%VO_2_peak (95% CI, 58%–65%VO_2_peak) during LC and 63% VO_2_peak (95% CI, 59%–66% VO_2_peak) during AC. The electronic workloads used in LC were 38 ± 7 W at low intensity and 116 ± 16 W at moderate intensity. Although significant interaction effects (environment × intensity × time) were observed for VO_2_, %VO_2_peak, and VCO_2_ (*p* < 0.001), *post‐hoc* tests did not show significant pairwise differences between LC and WC at all time points.

**TABLE 1:** Expiratory gas variables, hemodynamic, and body temperature during land‐based (LC) and aquatic (AC) cycling at rest and during low to moderate intensity.

		LC				AC				*P*‐value			
		Rest		Exercise		Rest		Exercise		Main effects			Interaction
		Mean	(95% CI)	Mean	(95% CI)	Mean	(95% CI)	Mean	(95% CI)	Enviro	Intensity	Time	
VO_2_ (mL/kg/min)	Low	3.8	(3.5,4.2)	10.6	(8.5,12.6)	4.0	(3.6,4.3)	10.6	(8.6,12.6)	0.734	<0.001	<0.001	<0.001
	Moderate	3.7	(3.3,4.0)	24.2	(22.2,26.2)	3.5	(3.2,3.8)	24.9	(22.8,26.9)				
%VO_2_max (%)	Low	10	(8,11)	27	(23,31)	10	(9,12)	27	(24,31)	0.627	<0.001	<0.001	<0.001
	Moderate	9	(8,11)	61	(58,65)	9	(8,10)	63	(59,66)				
VCO_2_ (mL/kg/min)	Low	3.3	(2.9,3.6)	8.8	(6.8,10.9)	3.5	(3.2,3.9)	8.5	(6.4,10.5)	0.557	<0.001	<0.001	<0.001
	Moderate	3.1	(2.8,3.5)	23.2	(21.2,25.2)	2.9	(2.6,3.3)	24.8	(22.7,26.8)				
Heart rate (bpm)	Low	72	(61,83)	92	(82,103)	67	(56,78)	89	(79,100)	0.105	<0.001	<0.001	<0.001
	Moderate	67	(56,77)	131	(121,141)	57	(46,68)	133	(122,143)				
SBP (mmHg)	Low	114	(104,125)	135	(121,148)	102*	(91,113)	113*	(100,127)	0.001	<0.001	<0.001	<0.001
	Moderate	114	(103,124)	165	(152,179)	105*	(94,116)	171	(157,184)				
DBP (mmHg)	Low	77	(69,84)	72	(64,80)	60*	(52,67)	59*	(51,68)	<0.001	0.189	0.153	0.007
	Moderate	72	(65,79)	74	(66,82)	62*	(54,69)	74	(65,82)				
SV (mL)	Low	40	(24,55)	72	(51,93)	70*	(54,86)	85	(64,106)	0.001	<0.001	<0.001	<0.001
	Moderate	47	(32,63)	122	(101,143)	68*	(52,84)	132	(111,153)				
CO (L/min)	Low	2.9	(1.4,4.3)	6.6	(4.1,9.1)	4.9*	(3.4,6.3)	7.6	(5.1,10.1)	0.052	<0.001	<0.001	<0.001
	Moderate	3.1	(1.7,4.6)	16.0	(13.4,18.5)	3.9	(2.5,5.3)	17.3	(14.8,19.8)				
Body temperature (°C)	Low	35.3	(35.0,35.6)	35.2	(34.9,35.6)	35.5	(35.1,35.8)	35.6	(35.3,35.9)	0.001	0.891	0.034	0.175
	Moderate	35.2	(34.9,35.5)	35.3	(35.0,35.6)	35.3	(35.0,35.6)	35.9	(35.5,36.2)				

*Note*: Data are presented as mean and 95% CI. *p*‐values are calculated by the three‐way linear mixed model.

Abbreviations: AC, aquatic cycling; CO, cardiac output; DBP, diastolic blood pressure; LC, land‐based cycling; Low, low intensity; Moderate, moderate intensity; SBP, systolic blood pressure; SV, stroke volume; VCO_2_, carbon dioxide output; VO_2_, oxygen uptake; VO_2_max, maximal oxygen uptake.

* *p* < 0.05, versus land at the same intensity.

In hemodynamic variables, heart rates at all time points were not significantly different between the two environments. However, SBP and DBP were significantly lower during AC compared to LC, except at moderate‐intensity cycling. Additionally, SV in AC was higher at rest than in LC, but the difference was not statistically significant during exercise.

Body temperature showed a significant main effect of environment (*p* = 0.001).

### 3.2 Respiratory variables ventilatory equivalents to VO_2_ and VCO_2_

Table 2 shows respiratory variables during LC and AC. All respiratory variables changed as exercise intensity increased (main effect of intensity, all *p* < 0.05). Specifically, AC led to significantly higher V_E_ and RR (*p* = 0.004 and *p* = 0.003, respectively) and significantly lower Ttot (*p* = 0.014) at moderate intensity when compared to LC. In addition, V_T_ during AC was significantly lower at rest before moderate‐intensity cycling (*p* = 0.048).

**TABLE 2:** Respiratory variables during land‐based (LC) and aquatic (AC) cycling at rest and during low to moderate intensity.

	Intensity	LC				AC				*p*‐value			
		Rest		Exercise		Rest		Exercise		Main effects			Interaction
		Mean	(95% CI)	Mean	(95% CI)	Mean	(95% CI)	Mean	(95% CI)	Enviro	Intensity	Time	
V_E_ (L/min)	Low	9.5	(8.2,10.8)	19.8	(16.2,23.3)	9.6	(8.4,10.9)	19.4	(15.9,22.9)	0.067	<0.001	<0.001	<0.001
	Moderate	8.9	(7.6,10.2)	42.8	(39.2,46.3)	8.2	(6.9,9.5)	50.3*	(46.8,53.8)				
RR (bpm)	Low	18	(16,19)	24	(20,27)	18	(16,20)	25	(22,29)	0.005	<0.001	<0.001	<0.001
	Moderate	17	(15,19)	29	(25,32)	17	(15,19)	36*	(32,39)				
V_T_ (mL/breath)	Low	545	(490,600)	847	(713,980)	539	(484,594)	780	(647,914)	0.124	<0.001	<0.001	<0.001
	Moderate	538	(483,593)	1542	(1408,1675)	487*	(432,542)	1453	(1320,1587)				
Ttot (sec)	Low	3.53	(3.13,3.92)	2.59	(2.28,2.91)	3.58	(3.18,3.97)	2.44	(2.12,2.75)	0.148	0.027	<0.001	0.028
	Moderate	3.67	(3.28,4.07)	2.19	(1.87,2.50)	3.68	(3.29,4.08)	1.78*	(1.47,2.09)				
V_E_/VO_2_ (L/min/mL/kg/min)	Low	2.5	(2.2,2.8)	1.9	(1.7,2.1)	2.4	(2.1,2.8)	1.9	(1.6,2.1)	0.842	0.976	<0.001	0.001
	Moderate	2.4	(2.1,2.8)	1.8	(1.6,2.0)	2.4	(2.0,2.7)	2.1*	(1.8,2.3)				
V_E_/VCO_2_ (L/min/mL/kg/min)	Low	2.9	(2.6,3.2)	2.3	(2.0,2.5)	2.7	(2.4,3.0)	2.3	(2.1,2.6)	0.953	0.014	<0.001	0.003
	Moderate	2.9	(2.6,3.2)	1.9	(1.6,2.1)	2.8	(2.5,3.1)	2.1*	(1.8,2.3)				

*Note*: Data are presented as mean and 95% CI. *p*‐values are calculated by the three‐way linear mixed model.

Abbreviations: AC, aquatic cycling; LC, land‐based cycling; Low, low intensity; Moderate, moderate intensity; RR, respiratory rate; Ttot, total time per breath; V_E_, minute ventilation; V_E_/VCO_2_, ventilatory equivalent for carbon dioxide output; V_E_/VO_2_, ventilatory equivalent for oxygen uptake; V_T_, tidal volume.

* *p* < 0.05, versus land at the same intensity.

V_E_/VO_2_ and V_E_/VCO_2_ showed significant interaction of environment × intensity × time (*p* < 0.05). During AC at moderate intensity, V_E_/VO_2_ and V_E_/VCO_2_ were significantly higher than during LC at the same intensity (*p* < 0.001 and *p* = 0.004, respectively).

### 3.3 Lung volume variables

**FIGURE 2:**
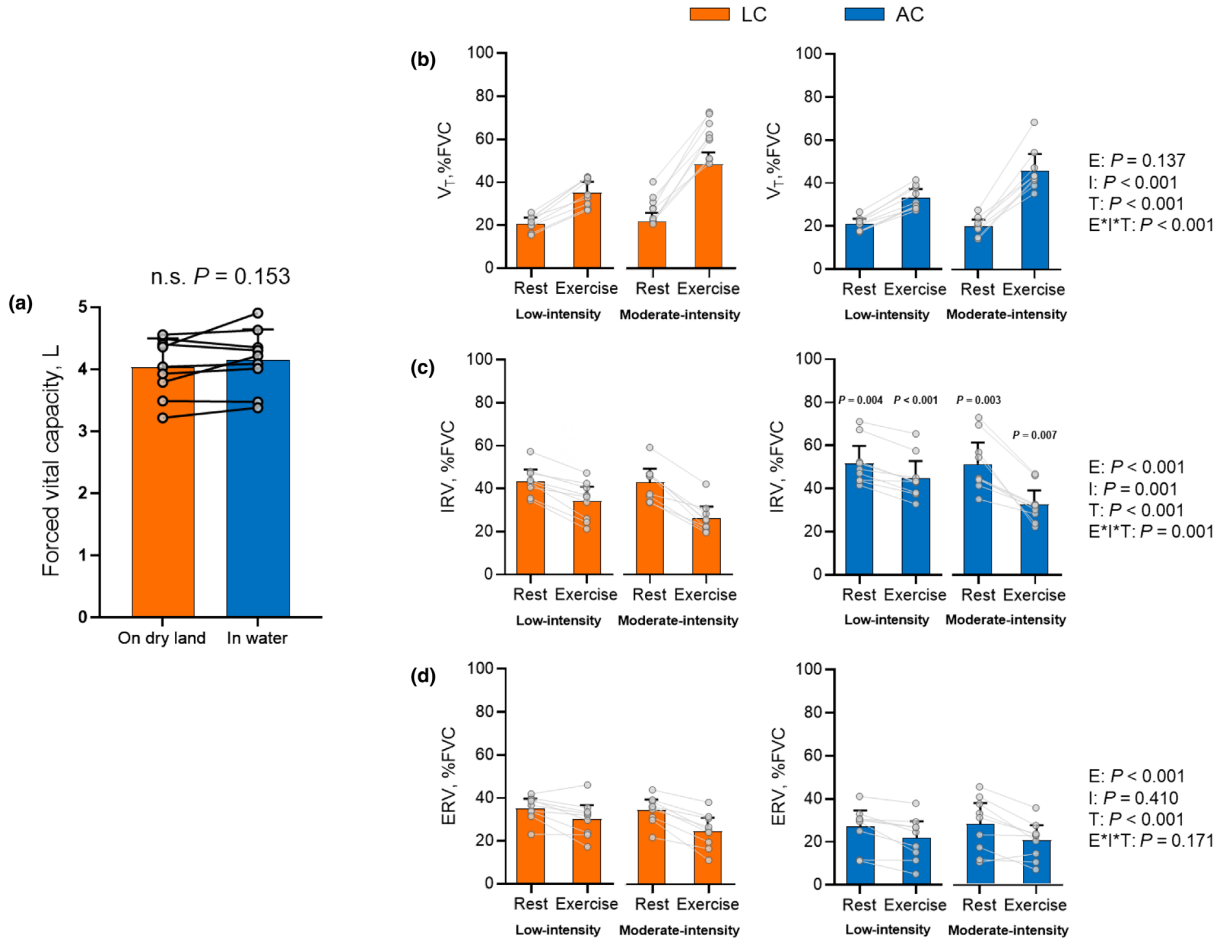
Forced vital capacity (FVC, [a]) tidal volume (V_T_, [b]), inspiratory reserve volume (IRV, [c]), and expiratory reserve volume (ERV, [d]) during land‐based (LC) and aquatic (AC) cycling. Participants (*n* = 9) performed land‐based (LC) and aquatic (AC) cycling. Orange and blue bars indicate the mean values measured in the LC and AC, respectively. Error bars indicate standard deviation for (b) and 95% CI for (b–d). *p*‐values are calculated by the three‐way linear mixed model for V_T_, IRV, and ERV and by paired t test for FVC based on the intent‐to‐treat analysis. Reported *p*‐values include main effects of environment (E), intensity (I), time (T), and their interaction (E*I*T). Significant *p*‐values compared between LC and AC at same intensity from post‐hoc tests are shown in the figure. ERV, expiratory reserve volume; FVC, forced vital capacity; IRV, inspiratory reserve volume; V_T_, tidal volume.

FVC and V_T_ did not differ significantly between environments (Figure 2a,b and Table 3). However, IRV was significantly higher (Table 3 and Figure 2c) and ERV was significantly lower (Table 3 and Figure 2d) during AC than LC (main effect of environment, all *p* < 0.001). In addition, IRV showed a significant interaction, and the results of the *post‐hoc* test confirmed that IRV was consistently higher during aquatic exercise when compared to LC at the matched intensity (Table 3 and Figure 2c).

**TABLE 3:** Lung volume variables during land‐based (LC) and aquatic (AC) cycling at rest and during low to moderate intensity.

		LC				AC				*p*‐value			
		Rest		Exercise		Rest		Exercise		Main effects			
		Mean	(95% CI)	Mean	(95% CI)	Mean	(95% CI)	Mean	(95% CI)	Enviro	Intensity	Time	Interaction
V_T_ (L)	Low	0.84	(0.73,0.95)	1.42	(1.25,1.59)	0.87	(0.76,0.98)	1.37	(1.19,1.54)	0.396	<0.001	<0.001	<0.001
	Moderate	0.88	(0.77,0.99)	1.96	(1.78,2.13)	0.82	(0.71,0.93)	1.90	(1.72,2.07)				
IRV (L)	Low	1.76	(1.43,2.10)	1.39	(1.06,1.71)	2.17*	(1.84,2.50)	1.88*	(1.56,2.20)	<0.001	0.002	<0.001	0.002
	Moderate	1.75	(1.42,2.08)	1.06	(0.74,1.39)	2.15*	(1.82,2.48)	1.37*	(1.05,1.69)				
ERV (L)	Low	1.42	(1.11,1.74)	1.22	(0.94,1.50)	1.11	(0.80,1.43)	0.90	(0.62,1.19)	0.005	0.538	<0.001	0.183
	Moderate	1.40	(1.08,1.72)	1.01	(0.72,1.29)	1.18	(0.86,1.50)	0.88	(0.60,1.16)				
V_T_ (%FVC)	Low	21	(18,24)	35	(31,40)	21	(18,24)	33	(29,38)	0.137	<0.001	<0.001	<0.001
	Moderate	22	(19,25)	49	(44,53)	20	(17,23)	46	(42,51)				
IRV (%FVC)	Low	44	(37,50)	34	(28,41)	52*	(45,59)	45*	(39,52)	<0.001	<0.001	<0.001	0.001
	Moderate	43	(36,50)	26	(20,33)	52*	(45,58)	33*	(26,39)				
ERV (%FVC)	Low	35	(28,43)	30	(24,37)	27	(20,34)	22	(15,28)	<0.001	0.410	<0.001	0.171
	Moderate	35	(28,42)	25	(19,31)	28	(21,35)	21	(14,27)				

*Note*: Data are presented as mean and 95% CI. *p*‐values are calculated by the three‐way linear mixed model.

Abbreviations: AC, aquatic cycling; ERV, expiratory reserve volume; FVC, forced vital capacity; IRV, inspiratory reserve volume; LC, land‐based cycling; Low, low intensity; Moderate, moderate intensity; V_T_, tidal volume.

* *p* < 0.05, versus land at the same intensity.

## 4 DISCUSSION

In this study, we conducted low‐ to moderate‐intensity exercise both in water and on land while matching VO_2_ between the two conditions. Although VO_2_ at each exercise intensity did not differ between the conditions, respiratory variables, including RR and V_E_, increased during moderate‐intensity exercise in water. Additionally, ventilatory equivalents relative to VO_2_ and VCO_2_ also increased. Furthermore, while there was no difference in FVC between both conditions, water immersion consistently led to a decrease in ERV and an increase in IRV both at rest and during exercise. We discuss the potential physiological mechanisms and clinical implications of these findings below.

**FIGURE 3:**
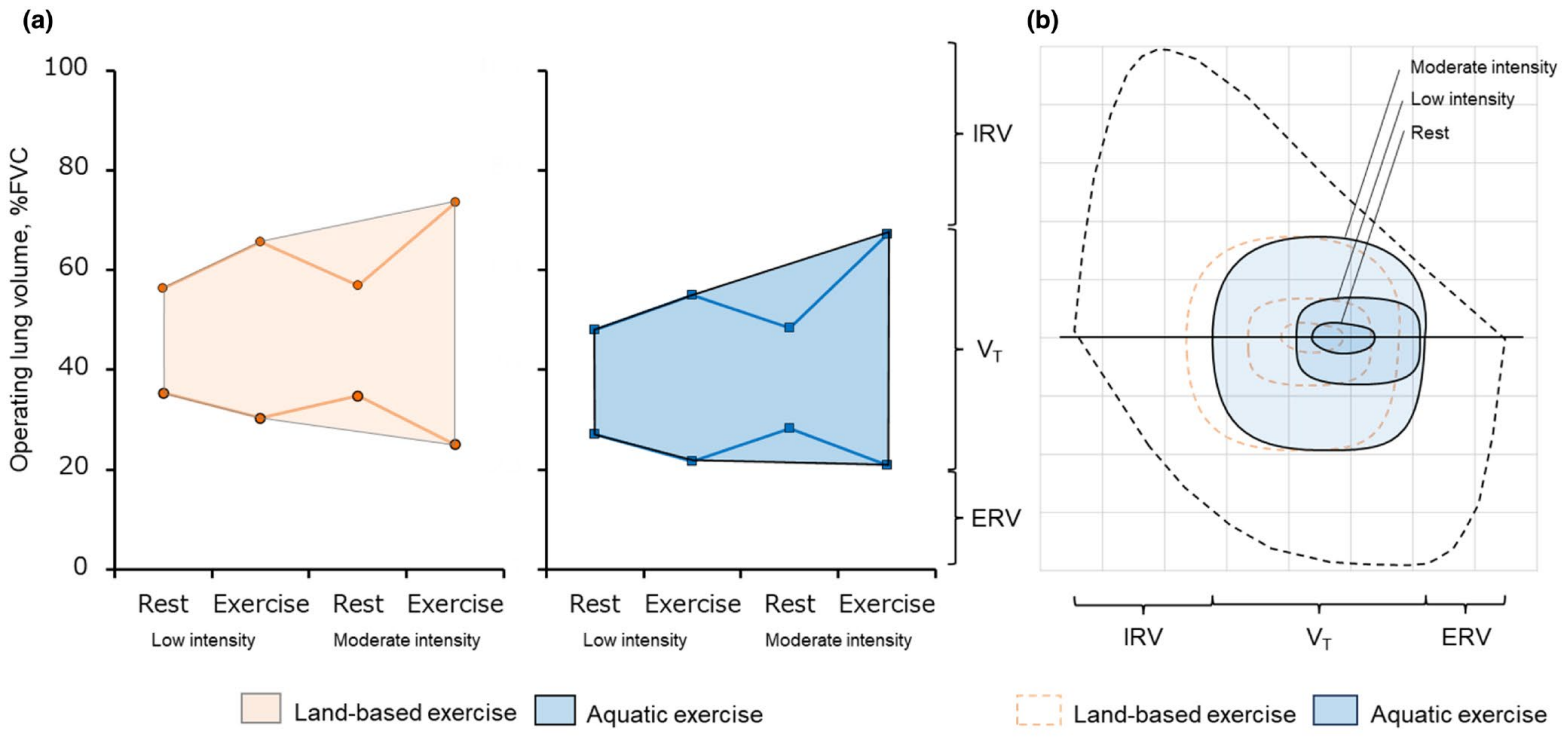
A hypothetical drawing to illustrate a displacement of operating lung volume (a) and flow‐volume loop (b) during aquatic exercise from rest to low to moderate intensity exercise compared to land‐based exercise. FVCs did not significantly differ in both environments; however, higher IRV and lower ERV during AC were maintained throughout rest to moderate intensity than the LC. Therefore, operating lung volume (a) and flow‐volume loop (b) during AC shifted downward and rightward, respectively.

During moderate intensity, V_E_ was significantly higher in AC than LC with increased RR, while there was no difference in V_T_ at moderate‐intensity exercise. These breathing patterns during AC are consistent with previous studies (Hoshi et al., 2022; Sheldahl et al., 1987) that concluded that increased RR during aquatic exercise may be caused by increased central blood volume and hydrostatic pressure on the thorax and abdomen due to water immersion. Consistently, ventilatory equivalents to VO_2_ and VCO_2_ were increased during moderate‐intensity aquatic exercise but not low‐intensity exercise in this study. In other words, moderate‐intensity aquatic exercise increases ventilatory demand, potentially leading to greater engagement of the respiratory muscles compared to land‐based exercise. This could serve as valuable evidence for determining exercise intensity in aquatic therapy for respiratory rehabilitation. Yamashina et al. reported a decrease in maximal respiratory muscle pressure after moderate‐intensity aquatic exercise (Yamashina et al., 2016). On the other hand, if respiratory muscle metabolism (i.e., VO_2_) increases to a greater extent during AC than during LC at this intensity, it is possible that the workload was actually lower in the aquatic condition compared to LC. As this study could not separately evaluate the load or VO_2_ of the respiratory muscles and lower limbs, future studies should ensure precise matching of oxygen demand and mechanical load. Water immersion may have altered the lung volumes at rest via increased venous return. The resting IRV increased by an average of 8% FVC and, conversely, the resting ERV decreased by 8% FVC due to water immersion compared to dry land condition. These results are consistent with previous studies that have examined ERV during immersion at the same water depth (Leal et al., 2010). Interestingly, the impact of water immersion on lung volume persisted during exercise, with increasing IRV and decreasing ERV observed at low and moderate intensity compared to LC. Previous studies have reported that lower ERV during water immersion is primarily due to decreased pulmonary compliance associated with increased venous return during water immersion (Agostoni et al., 1966; Bondi et al., 1976; Buono, 1983; Castagna et al., 2021). The averaged SV, a surrogate for venous return, tended to be higher during AC than LC at rest. Therefore, it is likely that exercise in water is performed at a lower lung volume compared to exercise on land (Figure 3).

Dynamic hyperinflation in patients with expiratory flow limitation such as chronic obstructive pulmonary disease is associated with dyspnea and one of the causes of decreased exercise tolerance (Gologanu, 2013; Kosmas et al., 2004; Stevens, 2018). The concept of operating lung volume (OLV) shows the dynamics between V_T_ and reserve volumes (IRV and ERV) during exercise and helps to understand the contribution of lung volume limitation for hyperventilation in patients (Guenette et al., 2013). In these patients, as exercise intensity increases, OLV deviates toward inspiration (i.e., decrease in IRV and increase in ERV) (Chuang, 2023). In the present study, the displacement of the OLV toward the expiratory side due to water immersion contrasts with the response seen in dynamic hyperinflation. In previous studies, it has been suggested that mechanical compression on the thoracic cavity decreases ERV, thereby improving dyspnea and peak VO_2_ during exercise (Ora et al., 2009, 2011). Although further investigation is needed in these patients, aquatic exercise may be an effective modality that reduces dynamic hyperinflation and dyspnea during exercise.

Hot‐ and cold‐water immersion have been shown to affect ventilation (Fujimoto et al., 2016; Tsuji et al., 2016). The water temperature in this study was strictly maintained at 31°C–32°C, which has been demonstrated to have the lowest effect on VO_2_ at rest and during exercise (Bréchat et al., 1999; Craig Jr. & Dvorak, 1966; McArdle et al., 1976). However, body temperature exhibited a significant main effect based on environment, with body temperature during AC being higher than LC, potentially influencing respiratory variables. Nonetheless, the difference in mean body temperature between both environments is negligible.

Several limitations are inherent in this study. Firstly, this study did not measure external power output during AC. The difference in external power output may have affected VO_2_ independently of the exercise environment (land vs. water). Moreover, the increase in V_E_/VO_2_ during moderate‐intensity AC suggests increased respiratory muscle metabolism, indicating that the load on the lower limbs may have been lower during AC compared to LC. Nevertheless, ventilation was higher and ERV remained consistently lower from rest to exercise in AC than in LC. This likely reflects the impact of aquatic exercise on the respiratory system. However, it was not possible to determine its exact contribution. Therefore, future studies comparing ventilatory responses between AC and LC should consider matching external power output in addition to metabolic rate. Secondly, while the sample size estimation was based on prior research, the small sample size may have led to diminished statistical power concerning other variables. Moreover, comparative analyses between men and women could not be conducted. Sex disparities in the respiratory system notably influence ventilatory responses during exercise. For instance, women typically exhibit lower lung capacities relative to height and age (Thurlbeck, 1982), as well as reduced respiratory muscle power (Aslan et al., 2019; Vincken et al., 1987) when compared to men. Additionally, lower V_T_ (%FVC) and maximal V_E_ during exercise in women may result in a shallower breathing pattern compared to men (Schaeffer et al., 2014). While the statistical comparisons could not perform the effects of sex differences on ventilatory responses during aquatic exercise due to small sample size, further investigations into respiratory system response stratified by sex are warranted. Thirdly, the order of AC and LC was not randomized in our study. Due to the lack of electronic control over the water stationary bicycle's load, it was necessary to measure VO_2_ during AC and subsequently adjust VO_2_ during LC. In this study, the water level was set at the xiphoid process, and thus the lungs were not fully submerged. However, at this depth, hydrostatic pressure on the thoracoabdominal cavity, along with increased venous return, may have sufficiently reduced lung compliance. The findings of this study suggest that aquatic exercise at such immersion levels may still provide effective respiratory training.

## 5 CONCLUSIONS

Moderate‐intensity cycling in water elicited a greater ventilatory equivalent for oxygen uptake compared with VO_2_‐matched land‐based exercise, while VO_2_ and FVC at rest were unaltered by water immersion to the xiphoid process. Moreover, during aquatic exercise, lung volumes remained lower in ERV and higher in IRV than during land‐based exercise. These findings suggest that an aquatic environment may be more effective than land‐based exercise for training the respiratory system, possibly due to the greater hydrostatic pressure, which alters lung volume and places an increased load on the respiratory muscles.

## AUTHOR CONTRIBUTIONS

All authors were responsible for the conception and design of this work. Daisuke Hoshi, Marina Fukuie, and Tsubasa Tomoto performed experiments. Daisuke Hoshi analyzed data, prepared figures and tables, and drafted the manuscript. All authors edited and revised the manuscript, approved the final version of the manuscript, and agree to be accountable for all aspects of the work in ensuring that questions related to the accuracy or integrity of any part of the work are appropriately investigated and resolved.

## CONFLICT OF INTEREST STATEMENT

The authors declare that they have no potential conflict of interest.

## Data Availability

The data that support the findings of this study are available from the corresponding author upon reasonable request.
